# An Approach to the Evaluation of Persistent Hypereosinophilia in Pediatric Patients

**DOI:** 10.3389/fimmu.2018.01944

**Published:** 2018-09-03

**Authors:** Justin T. Schwartz, Patricia C. Fulkerson

**Affiliations:** Division of Allergy and Immunology, Department of Pediatrics, Cincinnati Children's Hospital Medical Center, University of Cincinnati College of Medicine, Cincinnati, OH, United States

**Keywords:** children, eosinophil, hypereosinophilia, differential diagnosis, epidemiology, diagnostic testing

## Abstract

Hypereosinophilia (HE) is currently defined by a peripheral blood absolute eosinophil count (AEC) of ≥1,500 cells/microL. Although mild blood eosinophilia (AEC 500–1,500 cells/microL) is observed relatively frequently within the pediatric population, persistent HE is uncommon and should prompt additional clinical evaluation. While the clinical manifestations and underlying etiologies of HE in adults have been well-characterized, there is a paucity of data on HE in children. Limited evidence suggests that many similarities between adult and pediatric HE likely exist, but some important differences remain between these populations. The evaluation of HE in children can be challenging given the broad differential diagnosis, which includes primary hematologic disorders and secondary eosinophilia in which the increased eosinophil levels are propagated by disease states that promote eosinophil production and survival. On the basis of the underlying etiology, clinical manifestations can range from benign, self-resolving elevations in the AEC to life-threatening disorders with the potential for significant end-organ damage. Given the broad differential diagnosis of HE, it remains essential to systematically approach the evaluation of unexplained HE in children. This review will discuss the differential diagnosis for pediatric HE, highlighting etiologies that are more prevalent within the pediatric population. Additionally, a summary of the epidemiology of pediatric HE will be presented, with focus on some of the differences that exist between pediatric and adult HE. Finally, a directed approach to the diagnostic evaluation of children with HE will be discussed.

## Introduction

Eosinophils are terminally differentiated granulocytes with important roles in innate immune function, tissue remodeling and repair, and disease pathogenesis (1). The eosinophil level in the peripheral circulation is tightly regulated, with eosinophils representing only a small minority (typically <5–6%) of the circulating leukocyte population. Although commonly assessed in the peripheral blood, eosinophils are primarily tissue-dwelling cells (2). Under normal homeostatic conditions, the vast majority of eosinophils leave the circulation and migrate into specific tissues where they can reside for several weeks. The gastrointestinal tract serves as the largest tissue reservoir for eosinophils, where these cells are normally present in the mucosal tissue from the stomach to the large intestine (3, 4). Eosinophilia, denoted by an increased absolute eosinophil count (AEC) in the blood, can be driven by a number of important disease states in children and reflects the balance between eosinophil production in the bone marrow, trafficking from the bone marrow into the tissues and eosinophil apoptosis. Transient eosinophilia is observed relatively frequently in the pediatric population and is generally clinically insignificant. However, patients with chronic (persistent) eosinophilia can have a spectrum of clinical consequences, ranging from relatively benign disorders to disease states associated with significant end-organ dysfunction and potentially life-threatening sequelae. Defining the underlying pathology propagating eosinophilia is an essential first step in the management of pediatric hypereosoinophilia (HE) in order to tailor an appropriate treatment strategy. In this review, we will discuss the differential diagnosis for HE in children, focusing on etiologies that are more prevalent in the pediatric population and epidemiologic differences between children and adults. Additionally, we will present a directed approach to the diagnostic evaluation of pediatric HE, highlighting some important red flags that should prompt medical providers to pursue more intensive evaluation.

## Terminology

The AEC represents the frequency of circulating eosinophils in the peripheral blood (in cells per microliter [cells/microL]). Eosinophil levels in the peripheral blood vary by age, with higher upper threshold limits seen in infants and toddlers compared to adolescents and adults (5–7). However, for most children >2 years of age, an AEC value of >700 cells/microL is considered abnormally elevated. The severity of eosinophilia has been arbitrarily classified into mild (AEC from the upper limit of normal to 1,500 cells/microL), moderate (AEC 1,500–5,000 cells/microL) and severe (AEC>5,000 cells/microL) (4, 8). Eosinophilia can be transient, episodic or persistent (chronic). The term HE has been reserved for patients with moderate-to-severe persistent blood eosinophilia, defined as a blood AEC ≥1,500 cells/microL obtained on at least 2 separate occasions (interval ≥1 month) or marked tissue eosinophilia (4). Tissue HE can be defined as a percentage of eosinophils that exceeds 20% of all nucleated cells in the bone marrow or tissue infiltration that is deemed extensive by a pathologist (4). In situations where eosinophils are not directly observed within the tissue, the histologic evidence of extracellular deposition of eosinophil-derived granule proteins (ex. major basic protein, eosinophil peroxidase or eosinophil-derived neurotoxin) in the tissue can also be used as surrogate markers of tissue eosinophilia (9). Finally, the term hypereosinophilic syndrome (HES) is an umbrella term describing a heterogeneous group of disorders that are characterized by HE and evidence of end-organ damage or dysfunction directly attributable to tissue eosinophilia (10). From a practical standpoint, it remains important to recognize clinically that end-organ manifestations of eosinophilia may not manifest when the HE is first noted, with some individuals developing signs of organ dysfunction years after the HE initially presents (11).

## Causes of hypereosinophilia in children

**Figure 1:**
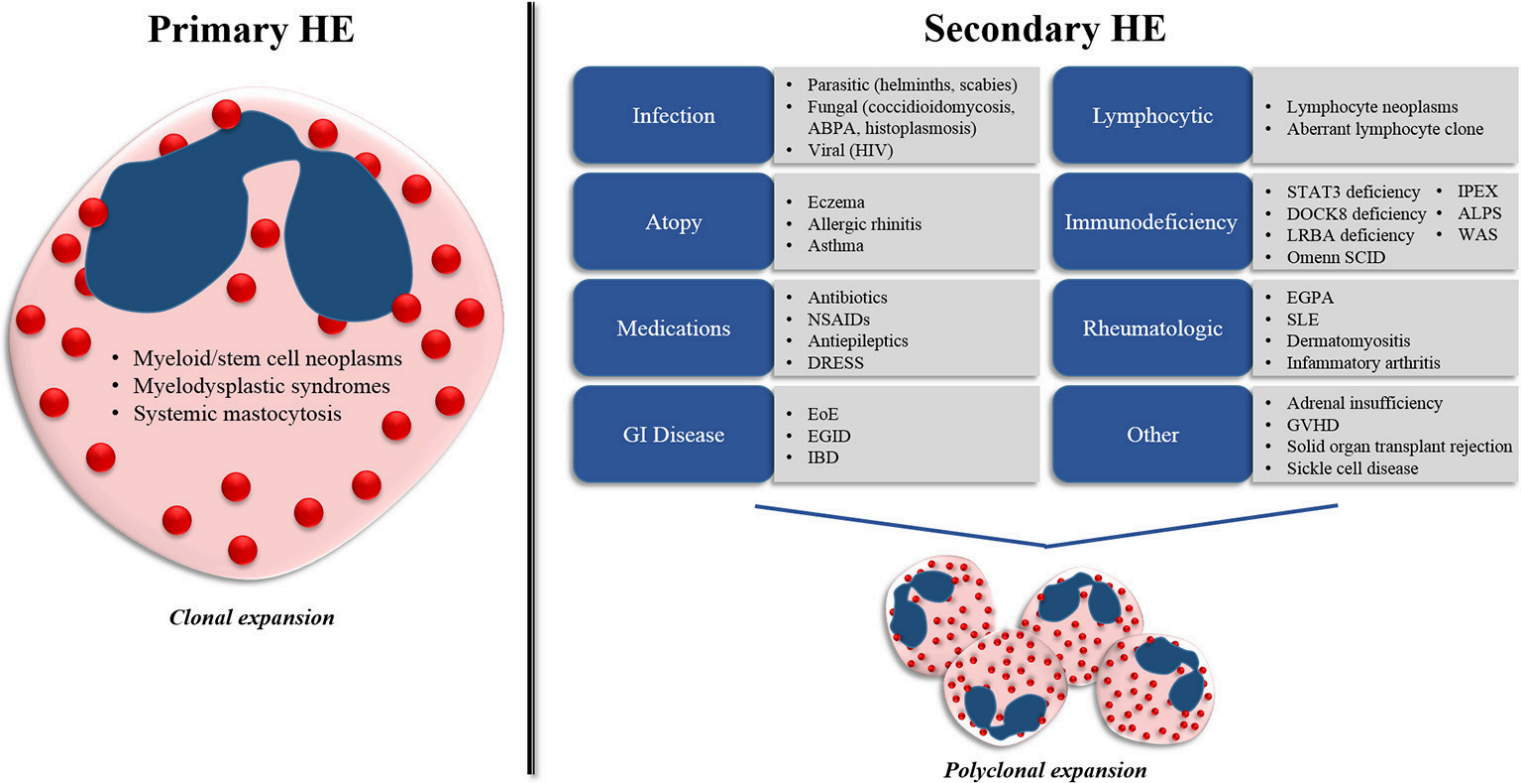
Differential diagnosis for hypereosinophilia (HE) in children. Pediatric HE can be separated into two main categories (primary and secondary HE) on the basis of the underlying mechanisms driving the eosinophil expansion. Primary HE results from myeloid and stem cell abnormalities that propagate the expansion of an eosinophil clone. Secondary HE results from a diverse group of disease states that drive the expansion of the eosinophil population through increased eosinophilopoietic cytokine (IL-3, IL-5, and GM-CSF) production. ABPA, allergic bronchopulmonary aspergillosis; HIV, human immunodeficiency virus; NSAIDs, nonsteroidal anti-inflammatory drugs; DRESS, drug reaction with eosinophilia and systemic symptoms; EoE, eosinophilic esophagitis; EGID, eosinophilic gastrointestinal disease; IBD, inflammatory bowel disease; STAT3, signal transducer and activator of transcription 3, DOCK8, dedicator of cytokinesis 8; LRBA, lipopolysaccharide-responsive-beige-like-anchor; WAS, Wiskott-Aldrich syndrome; IPEX, immune dysregulation, polyendocrinopathy, enteropathy, X-linked syndrome; ALPS, autoimmune lymphoproliferative syndrome; EGPA, eosinophilic granulomatosis with polyangiitis; SLE, systemic lupus erythematosus, GVHD, graft-vs.-host disease.

The underlying differential diagnoses for HE in children is similar to that of adults, with some notable exceptions. Pediatric HE can be associated with a variety of underlying etiologies that can be separated pragmatically into two main categories (primary and secondary HE) based on the underlying mechanisms driving the eosinophil expansion (Figure 1.)

### Primary (clonal) HE

Primary HE results from abnormalities within the bone marrow compartment that propagate the expansion of an eosinophil clone. In these disorders, eosinophils represent the predominant cell type involved or one of several proliferating cell lines. Primary causes of HE include myeloid and stem-cell neoplasms, grouped collectively into the category of myeloproliferative HE/HES (M-HE or M-HES) (10). This group of disorders includes both definitive and presumed eosinophilic myeloproliferative neoplasms, including hematopoietic neoplasms with eosinophilia resulting from fusion genes or mutations leading to the constitutive activation of oncogenic tyrosine kinase receptors such as PDGFRA, PDGFRB, or FGFR1, eosinophilic leukemia, myeloid leukemia, mast cell leukemia, myelodysplastic syndromes and systemic mastocytosis. Primary causes of HE represent a minority of pediatric HE cases (12, 13).

### Secondary (reactive) HE

Secondary (or reactive) HE results from disease states that drive the polyclonal expansion of eosinophils, typically through the increased production of cytokines such as IL-3, IL-5, and GM-CSF that promote increased eosinophil production and survival (14). Most cases of pediatric HE have an underlying secondary etiology.

### Infection

Normal immunological responses to certain infectious pathogens remain among the most common etiologies of secondary HE in children worldwide. In particular, invasive helminth infections (Strongyloidiasis, Schistosomiasis, Hookworm, Filariasis, Ascariasis, Toxocariasis, and Trichinosis) can cause pronounced eosinophilia early in infection as the larvae migrate through tissues (15). Some parasites are endemic worldwide and should be considered in all children with eosinophilia, regardless of their travel or exposure history. For example, *Strongyloides stercoralis* is endemic in areas with hot, humid climates (including the southeastern United States) and can directly penetrate the skin upon contact with soil or water contaminated with human feces (15). This parasite can have a long latency period of years between the initial exposure and symptom development, so an infection can easily be missed if the clinician does not routinely test for this infection (16). *Toxocara canis* and *cati* (the etiologic causes of visceral larva migrans) are also endemic worldwide and can be ingested in soil or food contaminated by dog or cat feces (15). Toxocariasis disproportionately affects the pediatric population, particularly toddler-aged children given their unsanitary ingestion habits (15, 17, 18). This parasite should be considered in any young child with HE, particularly if the child has a history of pica as these children are at increased risk for ingesting contaminated soil (17, 18). Nonhelminth infections can also trigger HE in the pediatric population. Scabies mite infection should be considered in children who present with a concomitant pruritic skin rash (19). Fungal etiologies, including allergic bronchopulmonary aspergillosis (ABPA), which can be seen in children with chronic lung disease (asthma, cystic fibrosis), and disseminated Coccidioidomycosis and Histoplasmosis infections can trigger HE (20–23). Finally, HIV is a rare cause of HE in the pediatric population and should be considered in patients with unexplained HE and risk factors (24–26). Notably, protozoal parasites that often affect the pediatric population (*Giardia, Cryptosporidium, Entamoeba*) generally do not produce peripheral HE (3, 15).

### Immune disorders

Atopic disease (eczema, allergic rhinitis, asthma) is a common cause of mild-to-moderate eosinophilia in the pediatric population and a minority of these patients can meet criteria for HE. However, the presence of severe, persistent eosinophilia (i.e., eosinophils >5,000/microL) is unlikely secondary to atopy and should prompt additional evaluation for another etiology. Importantly, several immunodeficiency syndromes (signal transducer and activator of transcription 3 [STAT3] deficiency, dedicator of cytokinesis 8 [DOCK8] deficiency, lipopolysaccharide-responsive-beige-like-anchor [LRBA] deficiency, Wiskott-Aldrich syndrome [WAS], and immune dysregulation, polyendocrinopathy, enteropathy, X-linked [IPEX] syndrome) present in childhood with atopy, elevated IgE and peripheral blood eosinophilia (27). Consequently, a thorough infection history (with focus on the frequency and etiology of infections) should be obtained in any pediatric patient with HE and atopy. Other immunodeficiencies that can present with HE in the pediatric population include autoimmune lymphoproliferative syndrome (ALPS) and Omenn syndrome associated with severe combined immunodeficiency (27).

### Drug hypersensitivity

Drug hypersensitivity reactions are also a frequent cause of HE in children. Antibiotics (particularly penicillins, cephalosporins, and vancomycin), NSAIDs, and antiepileptic medications are commonly implicated as causes of eosinophilia, but almost any prescription or nonprescription drug, herbal remedy, or dietary supplement can be a trigger (16, 28, 29). A temporal relationship between drug initiation and development of eosinophilia is helpful in identifying a drug reaction, although latency between exposure and eosinophilia can vary from days to months. Notably, drug reaction with eosinophilia and systemic symptoms (DRESS) is a potentially life-threatening systemic hypersensitivity reaction associated with peripheral HE that typically presents after a latency period of 2–8 weeks between drug exposure and clinical manifestations (fever, malaise, lymphadenopathy, elevated liver enzymes and morbilliform skin eruption that can progress to an exfoliative dermatitis) (30).

### Gastrointestinal disorders

Eosinophilic esophagitis (EoE) is also a common cause of HE in the pediatric age group. This diagnosis can often be missed if an appropriate history is not obtained. The primary symptoms of EoE vary with age, with younger patients presenting with feeding difficulties, frequent vomiting, food refusal/selective eating, and failure to thrive (31). As these children increase in age, complaints of abdominal pain and dysphagia increase and adolescents can develop food impactions. Other primary gastrointestinal eosinophilic disorders (eosinophilic gastrointestinal disease) can also cause HE in children, although these disorders are less common than EoE. Additionally, inflammatory bowel disease can be associated with a peripheral eosinophilia.

### Neoplasms

Lymphocytic variant HE/HES (L-HE or L-HES) is a category of disorders characterized by a clonal or aberrant lymphocyte population that produces cytokines that propagate eosinophil production and survival (10). Included within this category are lymphoid neoplasms, some of which are more common in children (ex. pre-B cell acute lymphoblastic leukemia [ALL]). ALL can present with HE in children, in some cases months before the underlying malignancy is detected (32, 33). In these situations, eosinophils are not part of the neoplastic clone and represent a secondary response to the malignancy.

### Autoimmune disorders

Less common etiologies of secondary HE in children include rheumatologic disease. Notably, eosinophilic granulomatosis with polyangiitis (EGPA, previously called Churg-Strauss syndrome) is a potentially life-threatening vasculitis rarely seen in the pediatric population and is commonly associated with moderate-to-severe peripheral blood eosinophilia, allergic rhinitis, and asthma. The most commonly involved organs include the lung and skin, although this disease can affect virtually any organ system, including the cardiovascular, gastrointestinal, renal, and central nervous systems (34). Other autoimmune diseases associated with HE in children include systemic lupus erythematosus, dermatomyositis, and inflammatory arthritis.

### Other

Adrenal insufficiency has been associated with eosinophilia, possibly due to the loss of endogenous glucocorticoids. Other secondary causes of HE to consider in certain clinical situations include graft-vs.-host disease following hematopoietic stem cell transplantation, solid-organ transplant rejection, and sickle cell disease.

## Epidemiology

The overall prevalence of HE within the pediatric population remains unknown as no population-based studies have been completed. Several retrospective cohort studies have focused on characterizing HE within the adult population (12, 35). However, a paucity of data on this subject exists within the pediatric literature, which is largely composed of single case reports and a small number of case series (36–38). Consequently, very little data exist with regards to the clinical presentation, underlying etiology, and prognosis of HE in the pediatric population and how it may differ from the adult population. Recently, Willams *et al*. published the largest retrospective cohort analysis comparing children and adults with HE. The study evaluated 291 patients (37 children, 254 adults) who presented to the National Institutes of Health for evaluation of unexplained HE between 1994 and 2012 (12). The most common diagnosis in both patient cohorts was idiopathic HES (46% of children and 47% of adults). A secondary cause for the HE was identified in only 14% of the children vs. 10% of the adults, with the most common etiology in both populations being helminth infection. Notably, all of the helminth infections in the pediatric cohort were *Toxocara* species compared to none in the adult cohort, providing evidence that an increased suspicion for *Toxocara* infection in children with HE is warranted. Primary immunodeficiency was noted more frequently in the pediatric vs. adult cohorts, although the number of cases was still limited (2/37 cases in pediatric cohort vs. 1/254 cases in adult cohort). In the patients that met criteria for HES, notable differences in the baseline characteristics in the pediatric population included male predominance, higher median peak AEC levels and higher median serum vitamin B12 levels. Clonal T-cell receptor rearrangement abnormalities were overrepresented in the adult population. The clinical manifestations were relatively similar between the two cohorts, with the exception of increased gastrointestinal involvement in children and increased pulmonary involvement in the adult cohort. Although the median peak AEC was almost twice as high in the pediatric cohort, mortality was low and similar to that in the adult cohort. Given that this study was compiled from a single-center, tertiary referral center (NIH), a referral bias is likely to exist within the study population, which thus may not completely reflect the true epidemiology of pediatric HE in the general population. Xiaohong *et al*. completed a retrospective analysis of the etiology of HE in 88 children admitted to the Children's Hospital of Zhejiang University School of Medicine in China between 2009 and 2015 (13). The most common etiology identified was infectious (parasite infections were the most common), followed by allergy and EGID. Immunodeficiency, hematologic neoplasms and EGPA were also noted in the study group. The limited amount of published data in children regarding the epidemiology and prognosis of HE makes definitive conclusions difficult; consequently, there remains a clear need for additional studies in this area to be completed.

## Clinical evaluation

Defining the underlying mechanism propagating a child's eosinophilia is an important first step in the management of pediatric HE, as effective therapy depends upon knowing whether to target the eosinophils themselves or a secondary condition that is driving eosinophil production. Given the broad differential for pediatric HE, a systematic diagnostic approach is necessary.

In general, the degree of eosinophilia is rarely useful for identifying the underlying cause of the eosinophilia, with the exceptions occurring at the extremes of the AEC spectrum (ex. persistent mild eosinophilia [500–1,500 Eos/microL] is more likely to be seen in atopic disease; severe eosinophilia [≥100,000 Eos/microL] is more likely to be caused by a myeloid neoplasm) (16, 39). Though much attention has focused on classifying patients on the basis of their blood AEC levels, organ dysfunction is ultimately caused by activated eosinophils infiltrating into the tissue, which is not always reflected by a concomitant increase in the AEC (16, 21). Conversely, patients with markedly elevated peripheral blood eosinophil counts may have little to no clinical symptoms (40). Consequently, the most important step in the initial evaluation of a child who presents with HE is to assess the presence and degree of illness symptoms, including signs of tissue/organ involvement. The urgency of the evaluation depends upon the acuity of the illness symptoms, the type of tissue/organ affected and the degree of organ dysfunction.

### History

All children with HE should undergo a thorough history to address symptoms indicating possible organ involvement, prior medical history, exposures (dietary, travel, medications), and prior eosinophil counts. Symptoms that may identify specific organ system involvement include fever, weight loss, fatigue, skin rash, nasal congestion, wheezing, cough, dyspnea, chest pain, dysphagia, vomiting, loss of appetite, abdominal pain, diarrhea and arthralgias/myalgias. Medical history, such as recurrent infections, atopy, inflammatory bowel disease, prior malignancy, or failure to thrive, can also be important when considering the differential diagnosis. Dietary history should include risk for ingestion of raw or undercooked meat, particularly wild game meat that can increase risk for Trichinosis. Ingestion of fruits, vegetables, or soil (i.e., a child with pica) possibly contaminated by dog or cat feces can be a risk factor for Toxocariasis. A history of travel to parasite-endemic areas may also suggest risk for other parasitic etiologies. As noted above, however, lack of travel or specific risk factors does not necessarily eliminate parasitic infection as some helminths are endemic worldwide and can have long latency periods (i.e., *Strongyloides*). Medication exposure is also important to evaluate, particularly in those children who are taking medications regularly and therefore have ongoing exposure. Finally, it is often useful to review prior AEC data if available. Chronic HE in the absence of symptoms is reassuring and suggests that the evaluation can be done less urgently if the child is otherwise healthy. When reviewing AEC trends, it is important to remember that several factors can transiently decrease eosinophil counts (i.e., steroids, bacterial or viral infections) causing the appearance of an eosinophilia that is waxing and waning.

### Diagnostic testing

**Figure 2:**
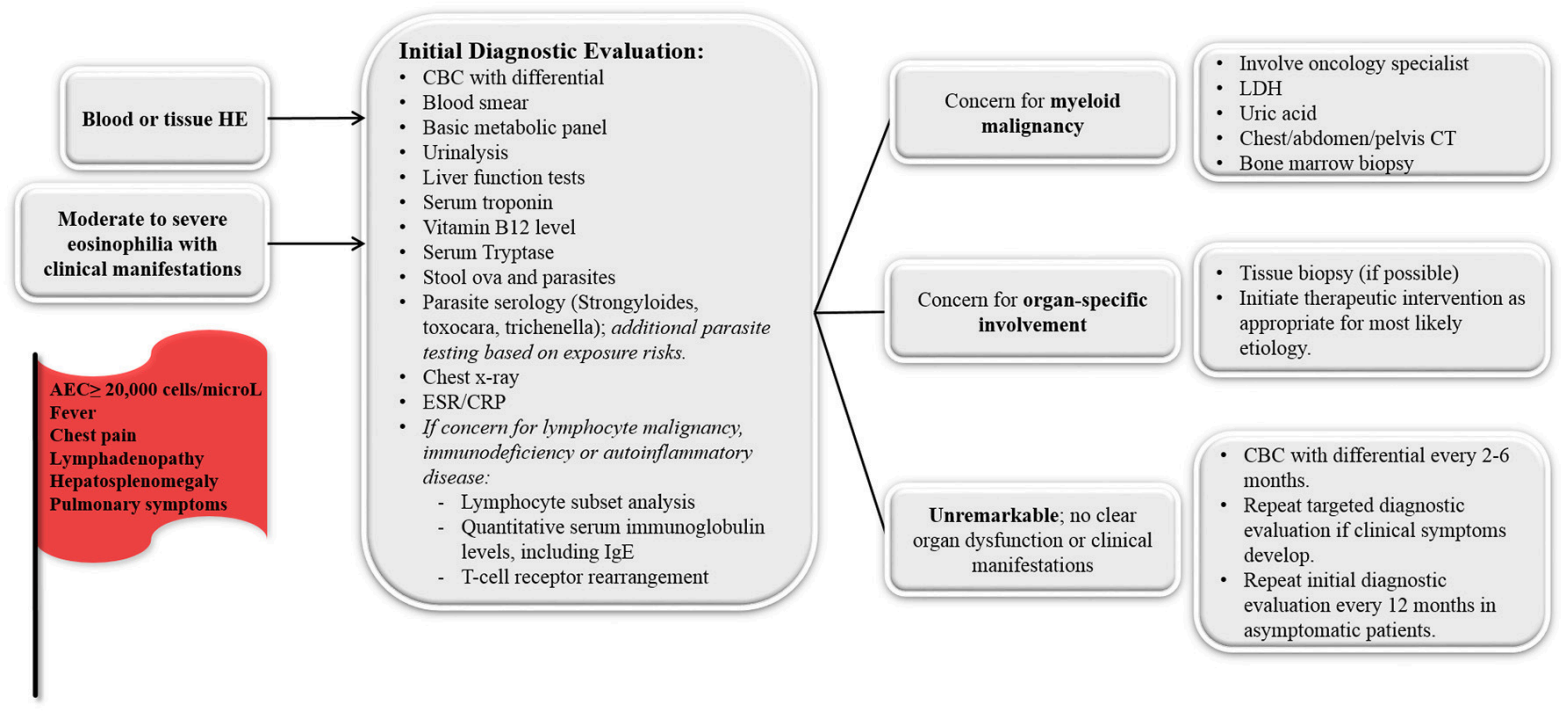
Diagnostic approach for the child who presents with unexplained hypereosinophilia (HE) and/or moderate-to-severe eosinophilia with clinical manifestations. Concerning symptoms/laboratory findings that should prompt medical providers to pursue more intensive evaluation are noted in the red flag. ESR, erythrocyte sedimentation rate; CRP, C-reactive protein; LDH, lactate dehydrogenase.

All children who meet diagnostic criteria for HE (i.e., blood AEC ≥1,500 cells/microL on at least 2 separate occasions [interval ≥1 month] or marked tissue eosinophilia) or moderate-to-severe eosinophilia with illness symptoms should undergo an initial diagnostic evaluation to try to determine the underlying etiology (Figure 2). A careful physical exam should be completed at every visit, noting any fever, nasal obstruction, abnormal or decreased lung sounds, skin rashes, abdominal tenderness, hepatosplenomegaly, lymphadenopathy or joint redness/swelling. Laboratory evaluation should include complete blood count with differential to evaluate for abnormalities in the other blood cell lines. A peripheral blood smear should be reviewed to evaluate for white blood cell blasts or other blood dyscrasias that could suggest a primary hematologic disorder. If blasts are noted, LDH, uric acid and hematology/oncology consultation is indicated. Bone marrow examination (aspiration and biopsy) should be considered for any child whose initial evaluation demonstrates no clear secondary etiology and a primary hematologic cause of the eosinophilia remains possible. In addition, bone marrow examination is appropriate for any acutely ill child with specific organ involvement and no clear underlying diagnosis, children with an eosinophil count >100,000 eosinophils/microL, or children with abnormal features on their peripheral blood smear (immature or dysplastic white blood cells, thrombocytopenia, or unexplained anemia). Serum chemistries, creatinine, and urinalysis should be completed to evaluate for evidence of renal or bladder involvement. Abnormal serum chemistries could also suggest underlying adrenal insufficiency. Liver function tests (to determine hepatic involvement) and cardiac troponin levels (for evidence of subclinical myocardial disease) should also be obtained. Patients with an elevated troponin levels should be further evaluated with electrocardiography and echocardiography. Serum B12 level should be obtained as a screening marker for myeloproliferative neoplasms and autoimmune lymphoproliferative syndrome (ALPS). Serum tryptase can be obtained to screen for systemic mastocytosis. Stool testing for ova and parasites and serologic testing for endemic parasites should also be routinely completed (*Strongyloides, Toxocara, Trichenella*). The indication for additional parasite testing is typically determined by exposure (diet, travel). Chest radiography should be completed to evaluate pulmonary involvement. Finally, in patients with a history of recurrent infections, lymphadenopathy, and/or hepatosplenomegaly, flow cytometry to evaluate lymphocyte subsets and immunoglobulin levels can be sent to screen for lymphocyte clonality and selective lymphocyte and immunoglobulin deficiencies. Additionally, T-cell receptor rearrangement studies can be useful to provide evidence of oligoclonality in the lymphocyte compartment. Finally, depending on risk factors, HIV testing may be indicated.

For those in whom the above evaluation is unremarkable and have no signs of organ involvement, repeat screening with a CBC with differential every 2–6 months to monitor AEC levels is a reasonable approach. If the AEC remains stable and the child remains healthy, repeating the above testing at 12-month intervals is appropriate. The development of new symptoms or an increasing AEC should prompt more immediate reevaluation.

### Exceptions for the acutely ill child with eosinophilia

Any child with acute illness symptoms (fever, evidence of end-organ dysfunction) and unexplained eosinophilia or a child with an extremely high blood eosinophil count (≥20,000 Eos/microL) requires hospitalization for immediate evaluation to determine the underlying cause. Notably, bacterial and viral infections typically cause a decrease in the blood eosinophil count, so the combination of fever and eosinophilia is an important red flag that should prompt consideration of other etiologies (15). Irrespective of the underlying etiology, the potential complications related to eosinophil tissue infiltration are similar (16). The most serious complications associated with HE are myocardial damage (i.e., myocarditis), pulmonary involvement and neurological involvement. Splinter hemorrhages and elevated serum troponin levels are indicative of cardiac involvement. Respiratory failure with pulmonary infiltrates are suggestive of pulmonary involvement. Neurological manifestations of HE include encephalopathy, sensory polyneuropathy and cerebral infarction. If evidence exists to suspect that the acute illness or organ dysfunction is secondary to tissue eosinophil infiltration, urgent therapy directed at reducing the eosinophilia (i.e., high dose glucocorticoids) is indicated (10). Patients with potential exposure to *Strongyloides* should receive concomitant empiric therapy with ivermectin to prevent corticosteroid-associated hyperinfection syndrome. In most situations, diagnostic laboratory testing to determine the underlying etiology of the eosinophilia should be obtained before initiating urgent empiric therapy, but treatment should not be delayed while awaiting the results of these studies.

## Conclusions

There is a paucity of data focused on HE in children, and we continue to have much to learn about the differences between HE in adults and children. The evaluation of HE in children can be challenging given the broad differential diagnosis and the wide range of clinical consequences, which include self-resolving elevations in the AEC and life-threatening disorders. Given the broad differential diagnosis of HE, it remains essential to systematically approach the diagnostic evaluation of unexplained HE in children.

## Author contributions

All authors listed have made a substantial, direct and intellectual contribution to the work and approved it for publication.

### Conflict of interest statement

PF has received grants from the National Institutes of Health, has served as a consultant for Genentech, Inc. and has received research funding from Knopp Biosciences, LLC. The remaining author declares that the research was conducted in the absence of any commercial or financial relationships that could be construed as a potential conflict of interest.
